# Plasma asymmetric dimethylarginine and its association with cardiovascular risk factors in Vietnamese patients before and after kidney transplantation, 2018–2022

**DOI:** 10.3389/fneph.2026.1773762

**Published:** 2026-06-11

**Authors:** Nguyen Thi Thuy, Le Viet Thang

**Affiliations:** 1 Department of Nephrology and Dialysis, Military Central Hospital 108, Ha Noi, Vietnam; 2 Department of Endocrinology, Military Central Hospital 108, Ha Noi, Vietnam; 3 Department of Nephrology and Dialysis, Military Hospital 103, Ha Noi, Vietnam

**Keywords:** asymmetric dimethylarginine, cardiovascular events, cardiovascular risk factors, dialysis, end-stage renal disease, kidney transplantation

## Abstract

**Background:**

Asymmetric dimethylarginine (ADMA) levels in patients with chronic kidney disease (CKD) are associated with cardiovascular events and mortality following kidney transplantation (KT). Therefore, ADMA may serve as a risk factor for cardiovascular complications in kidney transplant recipients. This study aimed to evaluate plasma ADMA concentrations and their association with cardiovascular risk factors in Vietnamese patients with end-stage renal disease (ESRD) before and after KT.

**Methods:**

From January 2018 to December 2022, 75 patients with ESRD who underwent KT were clinically evaluated using Doppler ultrasound, laboratory tests, and ADMA quantification both before and 6 months after KT. Additionally, 80 healthy people were selected as a control group. ADMA levels before and after KT were statistically analyzed to identify associations with cardiovascular risk factors.

**Results:**

The findings indicate that 100% of patients with ESRD before KT exhibited elevated levels of urea, creatinine, proteinuria, a long with a decreased glomerular filtration rate (GFR)< 60 ml/min. After KT, these abnormalities decreased to 17.3%, 41.3%, 5.3%, and 9.3%, respectively. Clinical indices such as hypertension, elevated CRP, anemia, left ventricular hypertrophy, and EF%< 50% were observed after KT at rates of 29.3%, 21.3%, 61.4%, and 34.7%, respectively. However, dyslipidemia and AIP > 0.11 increased by 2.6% and 10.7%, respectively, after KT compared to before. The average ADMA concentrations of patients before KT, after KT, and in healthy controls were 0.62 µmol/L, 0.57 µmol/L vs 0.17 µmol/L, respectively. Before KT, 93.3% of patients exhibited elevated ADMA levels, which decreased to 32.0% after KT. Males presented higher ADMA levels than females both before and after KT, as well as in healthy controls. ADMA levels showed minimal variation across different age groups. Before KT, dyslipidemia (cholesterol), AIP (> 0.11), and LA diameter were associated with increased ADMA levels. Lower hemoglobin and lower HDL-C levels at 6 months post-transplant were independently associated with an increased risk of ADMA elevation from pre-transplant to 6 months post-transplant.

**Conclusion:**

ADMA levels in patients with end-stage renal disease were 3.65 times higher than those in healthy controls. Although ADMA levels decreased after KT, they remained elevated compared to healthy controls. Hemoglobin and LDL-C were independent factors associated with changes in ADMA concentration between the pre-transplant and six-month post-transplant periods.

## 1 Introduction

Chronic kidney disease (CKD) is becoming a major global health issue and a significant burden on the healthcare system, particularly in low-income countries. As of 2022, more than 10% of the world’s population – over 850 million people - was reported to have CKD, with China and India accounting for the majority of cases (1, 2). End-stage renal disease (ESRD) represents the most severe stage of CKD, corresponding to stage 5 according to the American Society of Nephrology classification, characterized by a glomerular filtration rate (GFR)< 15 ml/min/1.73 m^2^ or the need of dialysis (3). Patients with ESRD face a substantially increased risk of mortality, primarily due to cardiovascular disease. Previous studies have found that patients with ESRD have a 10-100 folds higher risk of death from cardiovascular disease compared to healthy controls (4). Diabetes, hypertension, dyslipidemia, overweight/obesity, anemia, inflammation, and advanced age are all recognized as independent predictors of cardiovascular disease in ESRD patients (5). Between 1990 and 2021, the number of ESRD cases increased by more than 30%, with over 1.2 million deaths reported in 2021, ranking ESRD as the tenth leading causes of death worldwide. More than 7.6% of deaths were attributed to cardiovascular disease in conjunction with renal impairment, with ESRD accounting for 4.6% of these deaths (1, 6, 7).

Kidney transplantation is the best alternative treatment for ESRD because it allows patients to live a nearly normal life. However, pre-transplant cardiovascular complications persist and remain the leading cause of death in patients undergoing KT (8). Worldwide, the incidence of cardiovascular events in ESRD patients after KT is approximately 3.5 - 5%, which is 50 times higher than in healthy controls (9). Cardiovascular risk factors exist in patients prior to transplantation and are further influenced by the use of immunosuppressive therapies. Recent studies suggests that CRP, homocysteine, and asymmetric dimethylarginine (ADMA) are associated with cardiovascular events in kidney transplant recipients.

Plasma ADMA concentrations in patients with CKD are 1.13-1.36 times higher than in healthy controls, reaching their peak during the ESRD phase (10, 11). ADMA is a vasoconstrictor that inhibits nitric oxide (NO) synthesis. Its levels are inversely correlated to glomerular filtration rate and has been associated with cardiovascular events in patients both before and after kidney transplantation (12). Approximately 20% of ADMA is excreted in the urine by the kidneys, while the enzyme dimethylarginine dimethylaminohydrolase (DDAH) metabolizes the remaining 80% into citrulline and dimethylamine (13). Previous studies conducted in Belgium, Serbia, China, and Turkey have demonstrated that plasma ADMA concentrations significantly decreased after kidney transplantation compared to pre-transplant levels (14–16).

Elevated ADMA levels are associated with cardiovascular complications and increased mortality risk in patients with ESRD both before and after kidney transplantation. Therefore, ADMA may serve as a predictor of cardiovascular complications in kidney transplant recipients. Currently, there are very few studies in Vietnam examining ADMA and cardiovascular risk factors in post-kidney transplantation patients, and no studies investigating the correlation between cardiovascular risk factors and ADMA levels. The objective of this study is to describe the plasma ADMA levels in Vietnamese patients with ESRD before and 6 months after kidney transplantation, as well as to assess their association with several cardiovascular risk factors.

## 2 Methods

### 2.1 Patients and study design

From January 2018 to December 2022, 112 patients with ESRD underwent kidney transplantation; however, only 75 patients completed a full 6-month followed-up post-transplant and met the inclusion criteria for this study. The patients originated from 26 provinces and cities across Vietnam, including Bac Giang (7), Bac Ninh (5), Binh Phuoc (1), Da Nang (1), Dac Nong (1), Ha Giang (1), Ha Nam (1), Ha Noi (11), Ha Tinh (3), Hai Duong (4), Hai Phong (3), Hung Yen (2), Lam Dong (1), Lang Son (2), Lao Cai (4), Nam Dinh (4), Nghe An (5), Ninh Binh (1), Phu Tho (1), Quang Ninh (1), Thai Binh (3), Thai Nguyen (1), Thanh Hoa (4), Ho Chi Minh (2), Vinh Phuc (5), Yen Bai (1). Patients with ESRD underwent clinical examinations, laboratory tests, kidney transplantation, and comprehensive follow-up at Military Hospital 103 for six months. Furthermore, the study included 80 healthy controls with a similar male-to-female ratio and age distribution as a reference group. The reference group completed laboratory tests and had a single measurement of ADMA concentration.

### 2.2 Patient selection criteria

Patients diagnosed with ESRD according to KDIGO 2021 guidelines (3), > 18 years, who consented to participate in the study, underwent kidney transplantation surgery at Military Hospital 103. All patients received comprehensive clinical examinations and laboratory tests. Furthermore, patients underwent rigorous pre-transplant treatment, including optimal hemodialysis combined with antihypertensive and anemia therapies, as recommended by KDIGO 2021. Patients with ESRD who received a kidney transplant from a lawful source (a family member) or a brain-dead donor (excluding prisoners) were followed clinically and underwent laboratory testing six months post-surgery.

### 2.3 Patient exclusion criteria

Patients with malignancies such as Hodgkin lymphoma, non-Hodgkin lymphoma, and multiple lymphoma (Kahler disease), as well as those with acute inflammatory conditions such as pneumonia or acute viral infections, were excluded from the study. Additionally, patients who were pregnant or breastfeeding at the time of the study, those suffering from renal failure caused by diabetes mellitus or systemic lupus erythematosus, and kidney transplant recipients from prisoners were also excluded.

### 2.4 Criteria for selecting healthy controls

Healthy controls were individuals who underwent routine health checkups and were selected concurrently with participants with ESRD. They were over 18 years of age and had no history of chronic diseases such as liver, kidney, urinary tract, gallbladder, or cardiovascular conditions. Additionally, healthy controls were free of infections and musculoskeletal disorders, were not pregnant or breastfeeding, did not smoke or consume alcohol, and consented to participate in the study.

### 2.5 Pre-transplant treatment for ESRD patients

Among 75 patients with end-stage renal disease (ESRD), 62 underwent hemodialysis, 6 received peritoneal dialysis, and 7 were managed with medical treatment alone. All patients were administered antihypertensive medications, primarily calcium channel blockers, along with calcium supplementation for those with calcium deficiency. Additionally, erythropoietin-stimulating agents were given to all patients.

### 2.6 Clinical and laboratory criteria

- Body mass index (BMI) (kg/m2): underweight (< 18.5), normal (18.5 – 22.9), overweight, and obese (> 23) (17).
- Ejection fraction (EF%): normal (≥ 50%) or reduced (< 50%) (18).
- Left ventricular mass index (LVMI): left ventricular hypertrophy ≥ 115 g/m^2^ in men and ≥ 95 g/m^2^ in women (19).
- Atherogenic index of plasma (AIP): low risk of cardiovascular disease (≤ 0.11), medium risk of cardiovascular disease (0.11 - ≤ 0.21), high risk of cardiovascular disease (> 0.21) (20).
- Castelii’s risk index (CRI): cardiovascular risk (CRI – I > 4.0; CRI – II > 3.0) (21).
- Atherogenic Coefficient (AC): cardiovascular risk (AC > 2.0) (21).
- Urea index (mmol/l): normal (2.5 – 7.5), increased (> 7.5) (22).
- Creatinine index (µmol/l). normal (50 – 110), elevated (> 110) (23).
- Protein index (g/l): negative (60 - 80), positive (> 80) (24).
- CRP index (mg/l): normal (< 2), elevated (≥ 2) (25).
- Glomerular filtration rate (GFR): stage 1 renal failure (glomerular filtration rate ≥ 90 ml/min), stage 2 renal failure (glomerular filtration rate 60–89 ml/min), stage 3 renal failure (glomerular filtration rate 30–59 ml/min), stage 4 renal failure (glomerular filtration rate 15–29 ml/min), and stage 5 renal failure (glomerular filtration rate< 15 ml/min) (3).
- Anemia (hemoglobin g/l): normal (male ≥ 129, female ≥ 119), anemic (male< 129, female< 119) (26).
- Diabetes mellitus (mmol/l): normal (fasting blood glucose< 7.0, blood glucose at any time< 11.1), elevated (fasting blood glucose ≥ 7.0, blood glucose at any time > 11.1) (27).
- Hypertension (mmHg): normal (systolic blood pressure< 120 and diastolic blood pressure< 80), elevated (systolic blood pressure > 140 and diastolic blood pressure > 90) (28).
- Dyslipidemia: a disorder in one of the basic lipoprotein components including: total cholesterol (> 5.2 mmol/l), triglyceride (> 1.7 mmol/l), HDL-C (< 1.0 mmol/l), and LDL-C (> 2.6 mmol/l) (29).

### 2.7 Quantification of plasma ADMA

The specimen used for quantifying plasma ADMA concentration was venous blood collected in EDTA-anticoagulated tubes. Plasma was separated from blood components by centrifugation at 2,500 rpm/min for 10 minutes, then transferred to a cryotube using a Pasteur pipette and stored at -80 °C until analysis. For KT patients, blood samples were collected at two time points: T0 (before transplantation) and T6 (6 months after transplantation). For healthy controls, a single blood sample was collected. 50 µl of plasma from the test sample and 200 µl of the accompanying STD, CTRL1, and CTRL2 samples from the Immundiagnostik AG ADMA ELISA kit (Immudiagnostik AG, Stubenwald-Allee, Bensheim, Germany) were added to the corresponding wells on the ELISA plate according to the provided diagram. Then, 150 µl of DERBUF solution and 50 µl of DER solution were added to each well. The plate was sealed tightly and mix thoroughly by inverting it 10 times. The plate was shaken at 200 rpm for 45 minutes at room temperature. Next, the ELISA plate was gently centrifuged, and 250 µl of CODIL solution were added to each well. The plate was sealed tightly, mixed thoroughly, and shaken again for 45 minutes. Anti-ADMA antibody (AB antibody) was then added to each well, the plate was sealed tightly, and incubate at 2-8 °C for 16 hours. After incubation, all the solution were discarded from the wells, and the wells were washed with 250 µl of WASHBUF solution, repeating this washing step 5 times. Subsequently, 100 µl of CONJ solution were added to the wells, and the plate was shaken at room temperature for 1 hour. The solution was then completely discarded, and the wells were rinsed again with 250 µl of WASHBUF solution, repeating this rinsing step 5 times. Next, 100 µl of TMB (SUB) substrate were added to the wells, which were incubated at room temperature in the dark for 14 minutes. Immediately afterward, 100 µl of STOP solution were added to the wells, and the plate was mixed thoroughly by gently shaking. The absorbance was then read immediately on the ELISA reader at wavelengths of 450 nm and 620 nm. The ADMA concentration of the test samples was calculated based on the calibration curve constructed from the data of the control samples processed in the same batch.

Increased ADMA at 6 months (T6) was defined as a positive change in ADMA concentration from baseline, calculated as ΔADMA (T6 – T0) > 0. Patients were therefore categorized into two groups: those with increased ADMA (ΔADMA > 0) and those without an increase (ΔADMA ≤ 0).

### 2.8 Data analysis

Data were collected, entered, managed, and processed using SPSS version 20.0 and Stata 10.0 software. Qualitative variables were presented as frequencies and percentage, while quantitative variables were expressed as means and standard deviations for normally distributed data or as medians and interquartile ranges for non-normal distributed data. Comparison of percentage between two or more groups were performed using the Chi-square or Fisher-Exact Test. Percentage comparisons between two groups before and after treatment were conducted using the McNemar Test. Mean comparisons between two independent groups were performed using the Student’s t-test or Mann-Whitney U test, while comparisons among more than two independent groups were conducted using ANOVA or the Kruskal-Wallis test. Paired mean comparisons between two groups before and after treatment were analyzed using the paired-sample t-test or Wilcoxon signed-rank test. Pearson and Spearman correlation coefficients are used to assess the relationships between continuous variables, with |r| ≥ 0.7 (very strong correlation); 0.5 ≤ |r|< 0.7 (fairly strong correlation); 0.3 ≤ |r|< 0.5 (moderate correlation); |r|< 0.3 (weak correlation); positive r (positive correlation); negative r (negative correlation). Multivariate correlations between multiple variables are used to find the RR and calculate the Odds Ratio (OR). Data were collected, entered, managed, and processed using SPSS.

### 2.9 Ethical approval

The data is for the purpose of this research project only and will not be used for any other purpose. Personal information of the research subjects is encrypted and kept confidential in accordance with regulations. The study has been approved by the Ethics Committee for Medical Research of Military Hospital 103.

## 3 Results

The male-to-female ratio among patient with ESRD was 2.75, compared to 2.64 in healthy controls. Age distributions were similar between patients and healthy controls: ≤ 30 years (29.3% vs 30.0%), 31–40 years (41.3% vs 40.0%), 41–50 years (18.7% vs 20.0%), and > 50 years (10.7% vs 10.0%) (**Table 1**). BMI categories were aldo comparable:< 18.5 kg/m^2^ (18.7% vs 18.7%), ≥ 18.5–< 23.0 kg/m^2^ (61.3% vs 60.0%), and > 23.0 kg/m^2^ (10.7% vs 10.0%). Alternative kidney treatment methods among patients included medical treatment (9.3%), hemodialysis (82.7%), and peritoneal dialysis (8.0%). Immunosuppressive regimens for kidney transplant recipients comprised Tacrolimus + mycophenolate mofetil (MMF) + Prednisone (81.3%), Cyclosporine + MMF + Pred (16.0%), and Tacrolimus + mTOR + Pred (2.7%) (**Table 1**).

**Table 1:** Characteristics of patients with ESRD and healthy people in the study.

Characteristics	Patients with ESRD n = 75 (n, %)	Healthy people n = 80 (n, %)
Gender		
Male	55 (73.3)	58 (72.5)
Female	20 (26.7)	22 (27.5)
Year old (year)		
≤ 30	22 (29.3)	24 (30.0)
31 – 40	31 (41.3)	32 (40.0)
41 – 50	14 (18.7)	16 (20.0)
> 50	8 (10.7)	8 (10.0)
Mean ( $\bar{\text{X}}\pm \text{SD}$)	36.7 ± 9.3	36.6 ± 7.8
BMI (kg/m^2^)		
< 18.5	14 (18.7)	15 (18.7)
≥ 18.5 -< 23.0	46 (61.3)	48 (60.0)
≥ 23.0	15 (20.0)	17 (21.3)
Mean ( $\bar{\text{X}}\pm \text{SD}$)	20.8 ± 2.6	21.3 ± 2.4
Renal replacement therapy		
Medical treatment	7 (9.3)	–
Hemodialysis	62 (82.7)	–
Peripheral dialysis	6 (8.0)	–
Regimen after kidney transplantation		
Tacrolimus + MMF+ Pred	61 (81.3)	–
Cyclosporine + MMF+Pred	12 (16.0)	–
Tacrolimus +mTor+Pred	2 (2.7)	–

MMF, mycophenolate mofetil; Pred, prednisolone.

After KT, urea and creatinine levels decreased by 82.7% and 58.7%, respectively. The average urea and creatinine levels before KT were 22.6 mmol/L and 885.2 µmol/L, respectively, which decreased to 5.72 mmol/L and 102.5 µmol/L after KT. Prior to transplantation, all patients exhibited proteinuria and a GFR< 60 ml/min. Following transplantation, these rates dropped to 5.3% and 9.3%, respectively. The average GFR improved from 12 ml/min before KT to 72 ml/min afterward. The prevalence of diabetes increased from 0.0% to 4.0%, while hypertension decreased from 89.3% to 60.0%, the rates of overweight and obesity remained constant at20.0%. Dyslipidemia slightly increased from 78.7% to 81.3%, and elevated CRP levels decreased from 41.3% to 20.0%. Anemia prevalence declined significantly from 90.7% to 29.3%, whereas the AIP > 0.11 increased from 60.0% to 70.7%. The percentage of patients with EF%< 50% showed no significant change before and after KT. However, left ventricular hypertrophy decreased markedly from 58.7% to 24.0%, with the average left ventricular mass index reducing from 118.4 to 93.9 (**Table 2**).

**Table 2:** Characteristics of cardiovascular risk factors in patients with ESRD before and 6 months after kidney transplant.

Characteristics	Patients with ESRD		p
	Before KT^*^ (n, %)	After KT^*^ (n, %)	
Ure (mmol/L)			
Normal	0 (0.0)	52 (82.7)	NA
Increase	75 (100.0)	13 (17.3)	< 0.001^a^
Mean, ( $\bar{\text{X}}\pm \text{SD}$)	22.6 ± 3.14	5.72 ± 1.62	
Creatinin (µmol/L)			
Normal	0 (0.0)	44 (59.7)	> 0.05^a^
Increase	75 (100.0)	31 (41.3)	< 0.001^a^
Mean, ( $\bar{\text{X}}\pm \text{SD}$)	885.2 ± 31.3	102.5 ± 23.1	
GFR (ml/min)			
Normal	0 (0.0)	68 (89.7)	NA
Decrease< 60 ml/min	75 (100.0)	7 (9.3)	< 0.001^a^
(Median(quartile)	12 (9 – 17)	72 (65 – 86)	
Proteinuria			
Negative	0 (0.0)	71 (94.7)	NA
Positive	75 (100.0)	4 (5.3)	< 0.001^a^
Diabetes			
No	75 (100.0)	72 (96.0)	> 0.05^a^
Yes	0 (0.0)	3 (4.0)	NA
Hypertension			
No	8 (10.7)	25 (40.0)	< 0.01^a^
Yes	67 (89.3)	45 (60.0)	< 0.001^a^
Overweight and obesity			
No	60 (80.0)	60 (80.0)	> 0.05^a^
Yes	15 (20.0)	15 (20.0)	> 0.05^a^
Dyslipidemia			
No	16 (21.3)	15 (18.7)	> 0.05^a^
Yes	59 (78.7)	65 (81.3)	> 0.05^a^
Increased CRP > 2 mg/l			
No	44 (58.7)	60 (80.0)	< 0.01^a^
Yes	31 (41.3)	15 (20.0)	< 0.01^a^
Anemia			
No	7 (9.3)	53 (70.7)	< 0.001^a^
Yes	68 (90.7)	22 (29.3)	< 0.001^a^
Left ventricular hypertrophy			
No	31 (41.3)	57 (76.0)	< 0.001^a^
Yes	44 (58.7)	18 (24.0)	< 0.001^b^
Mean, median(quartile)	118.4 (88.7-148.1)	93.9 (77.5-109.2)	< 0.001^c^
AIP			
≤ 0.11	30 (40.0)	22 (29.3)	> 0.05^a^
> 0.11	45 (60.0)	53 (70.7)	> 0.05^a^
EF%			
Decrease< 50%	1 (1.3)	0 (0.0)	N/A
Mean, ( $\bar{\text{X}}\pm \text{SD}$)	63.9 ± 6.6	69.2 ± 4.3	< 0.001^b^

KT, Kidney transplantation, ^a^Mc Nemar test; ^b^paired-sample T test; ^c^Wilcoxon test.

Statistically significant differences were observed in ADMA concentrations among patient groups (before and after KT) and healthy controls. The average ADMA concentration was 0.62 µmol/L in patients before KT, 0.57 µmol/L after KT, and 0.17 µmol/L in healthy controls. Before KT, ADMA concentrations were elevated in up to 93.3% of patients, decreasing to 32.0% after KT. Males exhibited higher ADMA concentrations than females both before KT (0.65 µmol/L vs. 0.55 µmol/L) and after KT (0.57 µmol/L vs. 0.46 µmol/L) in the patient groups, as well as in the healthy controls (0.19 µmol/L vs. 0.17 µmol/L). ADMA concentrations did not differ significantly across age groups (**Table 3**).

**Table 3:** Characteristics of plasma ADMA levels in kidney transplant patients with ESRD and healthy people.

ADMA characteristics	Patients with ESRD		Healthy people (n = 80)	p
	Before KT^*^ (n = 75)	After KT^*^ (n = 75)		
ADMA (µmol/L) Mean, median(quartile)	0.62 (0.49-0.74)	0.57 (0.47-0.65)	0.17 (0.13-0.23)	< 0.001^a^
Min	0.27	0.12	0.09	
Max	1.16	1.00	0.37	
ADMA status				
No increase (n, %)	5 (6.7)	51 (68.0)	80 (100.0)	< 0.001^a^
Increase (n, %)	70 (93.3)	32.0 (32.0)	0 (0.0)	< 0.001^a^
Gender				
Male	0.65 (0.52-0.75)	0.57 (0.49-0.63)	0.19 (0.14-0.23)	< 0.01^a^
Female	0.55 (0.46-0.69)	0.46 (0.47-0.61)	0.17 (0.12-0.19)	< 0.01^a^
Year old (year)				
≤ 30	0.57 (0.45-0.73)	0.57 (0.41-0.69)	0.21 (0.15-0.26)	< 0.01^a^
31-40	0.63 (0.53-0.74)	0.57 (0.44-0.70)	0.18 (0.13-0.21)	< 0.01^a^
41-50	0,62 (0.50-0.76)	0.55 (0.40-0.67)	0.17 (0.14-0.20)	< 0.01^a^
> 50	0.67 (0.56-0.74)	0.50 (0.38-0.65)	0.18 (0.14-0.24)	< 0.01^a^

KT, Kidney transplantation, aMc Nemar test.

There was an association between dyslipidemia, AIP, and plasma ADMA concentrations in patients before KT. Specifically, the ADMA concentration in patients with dyslipidemia before KT was 0.64 µmol/L, compared to 0.54 µmol/L in patients without dyslipidemia. Additionally, patients with an AIP< 0.11 had an ADMA concentration of 0.53 µmol/L, whereas those with an AIP > 0.11 had a concentration of 0.66 µmol/L. Other factors, such as elevated CRP, anemia, obesity, hypertension, and the etiology of kidney failure, did not show significant variations in ADMA concentrations (**Table 4**).

**Table 4:** The relationship between ADMA concentration and clinical and laboratory features of patients with ESRD before kidney transplant.

Characteristics	ADMA (µmol/L) median (quartile)	p
Causes of kidney failure		
Chronic glomerulonephritis	0.62 (0.49 – 0.75)	> 0.05^a^
Chronic pyelonephritis	0.59 (0.51 – 0.71)	
Hemodialysis status		
Not yet undergoing	0.67 (0.53 – 0.76)	> 0.05^a^
Already undergoing	0.62 (0.49 – 0.72)	
Arteriovenous Fistula (AVF)		
Yes	0.625 (0.49 – 0.72)	> 0.05^a^
No	0.657 (0.52 – 0.75)	
Postvoid residual		
No	0.61 (0.48 – 0.75)	> 0.05^a^
Yes	0.66 (0.52 – 0.71)	
Hypertension		
Yes	0.62 (0.50 – 0.74)	> 0.05^a^
No	0.66 (0.46 – 0.80)	
Left ventricular hypertrophy		
Yes	0.63 (0.53 – 0.73)	> 0.05^a^
No	0.61 (0.47 – 0.74)	
*Dyslipidemia*		
*Yes*	*0.64 (0.52 – 0.76)*	*< 0.01^a^*
*No*	*0.54 (0.39 – 0.64)*	
Overweight and obesity		
Yes	0.68 (0.49 – 0.87)	> 0.05^a^
No	0.62 (0.51 – 0.69)	
Anemia		
Yes	0.62 (0.51 – 0.74)	> 0.05^a^
No	0.61 (0.41 – 0.88)	
Increased CRP		
Yes	0.63 (0.55 – 0.81)	> 0.05^a^
No	0.61 (0.46 – 0.70)	
*AIP*		
*< 0.11*	*0.53 (0.42 – 0.63)*	*< 0.001^b^*
*0.11-0.21*	*0.66 (0.55 – 0.74)*	
*> 0.21*	*0.68 (0.60 – 0.84)*	

^a^ Mc Nemar test.

^b^ paired-sample T test.

Statistical evaluation of cardiovascular risk factors prior to transplantation indicated that cholesterol levels, AIP, and LA diameter were positively correlated with patients’ ADMA levels. Specifically, ADMA concentration showed a moderate correlation with cholesterol levels (r = 0.207, p< 0.05), AIP (r = 0.359, p< 0.001), and LA diameter (r = 0.250, p< 0.01) (**Table 5**).

**Table 5:** Correlation of ADMA levels and cardiovascular risk factors of patients with ESRD before kidney transplantation.

Indices	ADMA (µmol/L)		Correlation coefficient
	r	p	
*Cholesterol (mmol/L)*	*0.207^a^*	*< 0.05*	ADMA = 0.032*Cholesterol + 0.502
LDL-C (mmol/L)	0.13^a^	> 0.05	–
*AIP*	*0.359^b^*	*< 0.001*	ADMA = 0.188*AIP + 0.597
LVDd (mm)	*0.193*	*< 0.05*	ADMA = 0.005*Dd + 0.356
LVDs (mm)	0.169	> 0.05	–
*LA diameter (mm)*	*0.250*	*< 0.01*	ADMA = 0.008*LA diameter + 0.387
EF%	- 0.065	> 0.05	–
LVMI	0.147	> 0.05	–
AIP	0.465	< 0.001	ADMA = 0.174*AIP + 0.027*Cholesterol + 0.008*LA diameter + 0.246
Cholesterol (mmol/L)			
LA diameter (mm)			

LVDd, ventricular end-diastolic diameter; LVDs, ventricular end-systole diameter; LA, left atrium; LVMI, Left ventricular mass index.

^a^ Spearman.

^b^ Pearson correlation.

Six months after kidney transplantation, analysis revealed a moderate positive correlation between ADMA levels and uric acid (r = 0.287, p< 0.05), AIP (r = 0.468, p< 0.001), CRI-I (r = 0.319, p< 0.01), CRI-II (r = 0.311, p< 0.01), and AC (r = 0.319, p< 0.01). In contrast, ADMA levels showed no significant correlation with hemoglobin (r = 0.157, p > 0.05), urea (r = 0.141, p > 0.05), or CRP (r = 0.099, p > 0.05) (**Table 6**).

**Table 6:** Correlation of plasma ADMA concentration with hematological and biochemical indices at 6 months after kidney transplant.

Indices	ADMA (µmol/L)		Correlation coefficient
	r	p	
Hemoglobin (g/L)	0.157	> 0.05	–
Ure (mmol/L)	0.141	> 0.05	–
*GFR (ml/min)*	*-0.261*	*< 0.05*	ADMA = 0.773 – 0.003*GFR
*Uric acid (µmol/L)*	*0.287*	*< 0.05*	ADMA = 0.001*uric acid + 0.379
*AIP*	*0.468*	*< 0.001*	ADMA = 0.275*AIP + 0.482
*CRI – I*	*0.319*	*< 0.01*	ADMA = 0.046*CRI-I + 0.358
*CRI – II*	*0.311*	*< 0.01*	ADMA = 0.056*CRI-II + 0.394
*AC*	*0.319*	*< 0.01*	ADMA = 0.046*AC + 0.404
CRP (mg/L)	0.099	> 0.05	–

Multivariable logistic regression analysis showed that lower hemoglobin and lower HDL-C levels at 6 months post-transplant were independently associated with an increased risk of ADMA elevation from pre-transplant to 6 months post-transplant with p< 0.05 (**Table 7**). In contrast, no significant associations were observed between ADMA change and creatinine, GFR, uric acid, D-dimer (Dd), or LVMI (p > 0.05) (**Table 7**).

**Table 7:** Multivariable logistic regression analysis of factors associated with increased ADMA 6 months after kidney transplantation.

Indices	Odds ratio (OR)	Confidence interval (CI 95%)	p
*Hemoglobin*	*0.95*	*0.91 – 0.99*	*< 0.05*
Creatinine	1.05	0.99 – 1.11	> 0.05
GFR	1.06	0.99 – 1.14	> 0.05
Uric acid	0.99	0.98 – 1.00	> 0.05
*HDL-C*	*0.06*	*0.01 – 0.85*	*< 0.05*
Dd	0.87	0.75 – 1.03	> 0.05
LVMI	1.02	0.99 – 1.05	> 0.05

## 4 Discussion

### 4.1 Clinical and laboratory indices change before and after kidney transplantation

Evaluating biochemical indices before and six months after KT, 17.3% of patients exhibited elevated urea levels, and 41.3% showed increased creatinine levels, while the proportion of patients with GFR< 60 ml/min decreased to only 9.3%. The kidneys are responsible for eliminating urea, creatinine, and other waste products; elevated levels of these markers indicate impaired kidney function. Furthermore, GFR is used to assess renal function after KT by monitoring residual urea and creatinine in the bloodstream. Our findings demonstrated that the proportion of patients with GFR< 60 ml/min declined from 100% to 9.3%, corresponding with the reductions in urea and creatinine concentrations. Consequently, six months after KT, the majority of transplanted kidneys function effectively with no evidence of rejection. Nonetheless, several recipients still exhibited reduced GFR and mildly elevated urea and creatinine levels six months post-transplant, which may result from viral infections such as BK virus or cytomegalovirus (CMV), excessive immunosuppressive therapy, metabolic disorders, or hypertension. In transplant recipients with diminished renal function, careful monitoring and appropriate treatment are essential, as both internal and external factors continue to pose a risk of graft rejection. Cleas, Freney, and Said all reported significantly improved GFR after KT compared to before KT (14, 30, 31). Therefore, diligent monitoring of blood urea and creatinine levels following the procedure is an effective method to assess changes in kidney function.

Cardiovascular risk factors identified in patients with ESRD in this study included hypertension, diabetes, BMI > 23, dyslipidemia, elevated AIP, increased CRP, anemia, smoking, and hyperuricemia. A comparison of these indicators before and six months after KT revealed a significant improvement in hypertension, anemia, elevated CRP, left ventricular hypertrophy, and hyperuricemia. Previous studies have reported that post-transplant patients exhibit a cardiovascular disease prevalence ranging from 3.5 to 5% and have nearly 50-folds increased risk of developing cardiovascular disease compared to healthy controls (8, 32, 33). After KT, patients remain at risk for cardiovascular disease due to factors such as hypertension, diabetes, dyslipidemia, smoking, and overweight or obesity. Therefore, it is crucial to carefully monitor these risk factors to effectively prevent cardiovascular complications, as recommended by KDIGO guidelines (34).

### 4.2 Association between ADMA concentration and clinical and laboratory parameters of patients with ESRD before kidney transplantation

Our findings showed that ADMA levels in patients undergoing hemodialysis and peritoneal dialysis were not significantly different from those in patients not receiving dialysis. Furthermore, there were no notable differences between the patients with residual urine and those with and without AVF. El Shahawy observed no change in ADMA levels between dialysis and non-dialysis patients (35). Zhang reported variations in plasma ADMA levels across different dialysis modalities, including peritoneal dialysis (PD), hemodiafiltration (HDF), and hemodialysis (HD), with HD exhibiting the lowest ADMA clearance efficiency (36). This explains the lack of a significant difference in plasma ADMA concentration between patients not receiving dialysis and those undergoing HD. Consequently, to reduce plasma ADMA levels, dialysis techniques such as PD and HDF should be preferred to enhance removal efficiency.

Our findings suggest that elevated plasma ADMA levels were not associated with factors such as hypertension, diabetes, anemia, or hyperuricemia prior to KT. However, a strong association was observed with dyslipidemia and AIP. Impaired kidney function often leads to dysregulation of lipid metabolism, accumulation of fatty components in the circulation, accelerated development of atherosclerosis, progression of cardiovascular disease, and kidney pathology. Studies demonstrates that the body produces approximately 300 µmol of ADMA daily; about 250 µmol is metabolized by DDAH, with the remainder excreted by the kidneys (11). Consequently, elevated blood ADMA levels are directly proportional to the progression of atherosclerosis and hypertension. Correlation analysis between ADMA with lipid components revealed a positive, albeit modest, association between cholesterol and increased ADMA levels. Previous studies found no evidence of a relationship between cholesterol and elevated ADMA concentrations (37–39). Lipid metabolism disturbances in patients with ESRD exhibit variations in blood lipid profile, which depend on multiple distinct factors.

Atherosclerosis is the primary cause of renal artery stenosis, and AIP is used to assess the extent of atherosclerosis. Our findings indicated that over 60% of subjects exhibited an increase in AIP beyond the normal range. Univariate and multivariate logistic regression analyses revealed a positive correlation between the ADMA levels and the AIP metric. Consequently, higher AIP values correspond to increased ADMA concentrations in the blood and are associated with the progression of chronic kidney impairment. Fernandez-Macias observed a similar association between AIP and serum ADMA concentrations in kidney transplant recipients (20). Furthermore, LDL-C is also a significant predictor of atherosclerosis progression (40). When LDL-C levels are elevated, the risk of myocardial infarction increases threefold compared to typical individuals (40). Multivariate correlation analysis of ADMA with AIP, cholesterol, and left atrial size demonstrated a strong association between these three variables with elevated ADMA levels. This suggests that patients presenting with all three risk factors simultaneously face a higher likelihood of cardiovascular events.

### 4.3 Changes in ADMA concentration with clinical and laboratory indices six months after kidney transplant

Our findings indicate that ADMA levels significantly decrease six months after KT, yet remain elevated compared to those in healthy controls. This is because ADMA is primarily metabolized by the enzyme DDAH and excreted through the kidneys. Following transplantation, most transplanted kidneys function effectively, as evidenced by increased GFR and substantial reductions in urea and creatinine levels. However, due to variable graft performance post-transplant, several recipients continue to exhibit elevated plasma ADMA levels. Data on cardiovascular risk factors suggest an increased incidence of dyslipidemia and atherosclerosis after transplantation. Therefore, patients who have undergone KT require ongoing monitoring and evaluation to promptly adjust therapeutic strategies, both to protect the transplanted organ and to restore renal function.

Analyzing the multivariate relationship between ADMA levels at six months post-transplant, it was observed that ADMA concentrations were negatively associated with GFR and positively correlated with AIP and uric acid indices, while showing no significant association with hemoglobin, urea, cholesterol, LDL-C, or CRP levels. The inverse relationship between ADMA levels and GFR is logical, as an increase in GFR enhances the kidney’s ability to clear ADMA, resulting in lower blood ADMA concentrations. Previous studies have demonstrated that a decline in GFR corresponds to an increase in blood ADMA levels, and vice versa (12, 14, 30, 41). Moreover, other studies have reported inconsistent findings regarding the relationship between cholesterol and elevated ADMA (14, 41, 42). Additionally, we found no association between ADMA levels and hemoglobin, urea, creatinine, or uric acid indices, suggesting that fluctuations in these parameters are not linked to change in ADMA concentration. The lack of association my be explained by the role of erythrocytes in storing ADMA; the rupture of red blood cells releases a significant amount of free ADMA, thereby promoting the degradation of methylated proteins (43, 44). Prior to kidney transplantation, the accumulation of asymmetric dimethylarginine (ADMA) in red blood cells was believed to inhibit erythropoietin expression, contributing to erythropoietin resistance and worsening anemia. In this study, six months after transplantation, the transplanted kidney demonstrated significant functional improvement, with approximately 91% of patients exhibiting a GFR > 60 ml/min/1.73 m^2^. This improvement led to reduced ADMA levels, as the transplanted kidney produced its own dimethylarginine dimethylaminohydrolase-1 (DDAH-1) enzyme. Consequently, anemia in post-transplant patients was significantly alleviated. Therefore, the lack of correlation between decreased hemoglobin levels and ADMA six months post-transplant is entirely expected.

### 4.4 ADMA concentrations change in predicting cardiovascular events in patients before and after kidney transplantation

Multivariate correlation analysis of laboratory indices at 6 months post-transplant and changes in ADMA concentration from pre-transplant to 6 months post-transplant, we observed that lower hemoglobin and lower HDL-C levels were independently associated with an increased risk of ADMA elevation after KT. Conversely, identifying factors present before KT that are independently correlated with increased plasma ADMA concentrations is also crucial for assessing and predicting cardiovascular events that may affect recipients, as well as the survival of the transplanted kidney. Elevated ADMA levels contribute to kidney damage and reduce nitric oxide (NO) production, increasing the risk of atherosclerosis and other related complications, thereby escalating cardiovascular disease. Consequently, increased ADMA concentrations demonstrate a causal relationship with impaired kidney function and provoke serious cardiovascular complications in patients. Oxidation of LDL-C acts as a stressor and diminished the effectiveness of DDAH and statins, leading to elevated ADMA synthesis. The difference in ADMA concentrations between the preoperative period and six months postoperatively can be attributed to several mechanisms. Yokoro et al. observed that lower hemoglobin concentrations were associated with higher ADMA levels in erythrocytes, but not in plasma (45). Since the lifespan of red blood cells is approximately 120 days (about three months), measuring plasma ADMA concentrations at different time points may reveal certain variations. Therefore, this represents one of the limitations of the present study.

Limitations of the study: changes in ADMA levels, along with clinical and paraclinical indicators in transplant patients, were measured only after six months. Additionally, the study was conducted at a single hospital. Therefore, the long-term effects of ADMA and cardiovascular risk factors in transplant patients cannot yet be fully assessed. Multicenter studies are needed to provide a more comprehensive understanding.

## 5 Conclusion

Plasma ADMA levels in Vietnamese patients with ESRD were 3.65 times higher than those in healthy controls. Although plasma ADMA levels decreased after KT compared to pre-transplant, they remained significantly elevated relative to healthy controls. Before transplantation, plasma ADMA levels were positive correlated with atherosclerosis, dyslipidemia, and enlarged left atrial diameter. At six months post-transplantation, plasma ADMA levels were associated with decreased GFR, increased cholesterol, and atherosclerosis. Hemoglobin and LDL-C were independent factors associated with the change in ADMA concentration between the pre-kidney transplantation period and six months post-transplantation.

## Data Availability

The raw data supporting the conclusions of this article will be made available by the authors, without undue reservation.
